# Global seroprevalence and distribution of Getah virus in domestic and wild animals: A systematic review and meta-analysis

**DOI:** 10.14202/vetworld.2025.3464-3475

**Published:** 2025-11-23

**Authors:** Ahmad Adebayo Irekeola, Rafidah Hanim Shueb

**Affiliations:** 1 Basic Sciences Unit, School of Dental Sciences, Health Campus, Universiti Sains Malaysia, Kubang Kerian, Kota Bharu, Kelantan, Malaysia; 2 Microbiology Unit, Department of Biological Sciences, College of Natural and Applied Sciences, Summit University Offa, Offa, Kwara State, Nigeria; 3 Department of Medical Microbiology and Parasitology, School of Medical Sciences, Health Campus, Universiti Sains Malaysia, Kubang Kerian, Kelantan, Malaysia

**Keywords:** alphavirus, domestic animals, Getah virus, meta-analysis, seroprevalence, wild animals

## Abstract

**Background and Aim:**

Getah virus (GETV), a mosquito-borne alphavirus of veterinary importance, has caused periodic outbreaks in domestic animals, especially in Asia. Although several studies have reported evidence of infection in animals, the overall global seroprevalence remains unclear. This study aimed to comprehensively synthesize available evidence on the worldwide seroprevalence of GETV in domestic and wild animals and identify epidemiological patterns across host types, regions, and detection methods.

**Materials and Methods:**

A systematic search of PubMed, Scopus, ScienceDirect, and Web of Science was conducted following Preferred Reporting Items for Systematic Reviews and Meta-Analyses 2020 guidelines, without temporal or regional restrictions. Eligible studies reporting serological detection of GETV antibodies in animal populations were included. Data were extracted and analyzed using a DerSimonian–Laird random-effects model, with subgroup analyses stratified by country, host category, sampling period, and diagnostic method. Heterogeneity was quantified using I^2^ statistics, and potential publication bias was assessed with funnel plots and Egger’s regression test.

**Results:**

Fifteen studies (n = 10,211 animals) met the inclusion criteria. The pooled global seroprevalence of GETV was 33.3% (95% confidence interval: 24.2–43.9; I^2^ = 98.65%, p < 0.001). Malaysia reported the highest seroprevalence (77.2%), followed by China (41.8%) and South Korea (26.4%). Domestic animals (34.0%) exhibited higher exposure than wild species (29.2%), with pigs (43.1%) and cattle (43.2%) recording the highest rates. Studies using virus-neutralization tests yielded higher estimates (47.3%) than those employing enzyme-linked immunosorbent assay (31.4%) or hemagglutination inhibition (7.3%). Meta-regression revealed study location and diagnostic method as significant sources of heterogeneity.

**Conclusion:**

This meta-analysis demonstrates that GETV is endemic among Asian animal populations, particularly domestic livestock, indicating substantial virus circulation across species. The findings underscore the need for enhanced veterinary surveillance, standardized serological testing, and One Health-oriented monitoring frameworks to detect and mitigate GETV transmission risks. The absence of data from Africa, Europe, and the Americas highlights an urgent need for geographically expanded research to better understand the virus’s global distribution and zoonotic potential.

## INTRODUCTION

Emerging and re-emerging infectious diseases continue to pose major challenges to global human and animal health, particularly those caused by zoonotic pathogens capable of crossing species barriers. Among these, arthropod-borne viruses transmitted by vectors such as mosquitoes have drawn significant attention due to their potential for rapid spread and their profound economic and health consequences [1, 2].

Getah virus (GETV), an enveloped, single-stranded, positive-sense RNA virus of the genus *Alphavirus* and family *Togaviridae* [3], exemplifies such emerging threats. Historically regarded as a relatively obscure pathogen, GETV has recently gained prominence following reports of outbreaks in domestic animals and serological evidence of exposure among various wild animal species, underscoring its growing epidemiological and veterinary relevance [4].

First isolated in 1955 from *Culex gelidus* mosquitoes in Malaysia, GETV has since been detected in multiple countries, including Russia, Japan, China, South Korea, Thailand, Vietnam, and India [4]. Its identification in diverse mosquito species demonstrates broad vector competence and suggests a high potential for interspecies transmission. The clinical manifestations of GETV infection vary widely among animal hosts. In horses, infection typically presents with fever, skin rash, hind limb edema, and lymphadenopathy, which can significantly affect equine health and the horse industry [5]. In pigs, the virus is associated with reproductive disorders, while in other animals it often causes subclinical or mild infections. However, the complete range of susceptible hosts and the full clinical spectrum of disease remain insufficiently characterized [4, 6].

Despite nearly seven decades since its discovery, the epidemiological understanding of GETV remains limited and fragmented. Although several serological investigations have reported its presence in domestic and wild animals, most studies were localized, descriptive, and lacked standardized methodologies, preventing reliable global comparison. The absence of harmonized data on host range, geographical distribution, and diagnostic performance has hindered accurate assessment of the virus’s true burden in animal populations. Moreover, surveillance activities have been concentrated almost exclusively in Asia, leaving potential transmission dynamics in other regions uncharacterized [4].

Of particular concern is the limited exploration of GETV’s zoonotic potential. While no confirmed human infections have been documented, serological evidence from several studies indicates possible human exposure to the virus [7–9]. This finding highlights the need for a One Health–oriented approach that integrates animal, vector, and human surveillance. However, systematic evidence synthesis on animal exposure, which forms the foundation for understanding spillover risk, remains unavailable. Similarly, variations in diagnostic tools, such as enzyme-linked immunosorbent assay (ELISA), hemagglutination inhibition (HI), and virus-neutralization techniques, have produced inconsistent estimates that complicate comparative interpretation.

In contrast to other arboviruses such as chikungunya and West Nile viruses, for which pooled seroprevalence estimates have informed risk modeling and policy development [10, 11], GETV lacks a comprehensive global synthesis. This gap in quantitative knowledge impedes informed risk assessment, early warning development, and prioritization of vector-borne disease surveillance. A consolidated meta-analysis is therefore essential to establish the global sero-epidemiological baseline for GETV and to identify critical host and regional determinants of infection.

This study aimed to systematically collate and analyze existing serological evidence of GETV infection in animal populations worldwide. Specifically, it sought to:

1. Estimate the pooled global seroprevalence of GETV among domestic and wild animals using meta-analytic techniques
2. Identify host-specific and geographical variations in GETV exposure across countries, animal species, and time periods
3. Evaluate the influence of diagnostic methods on reported seroprevalence estimates and explore sources of heterogeneity through subgroup and meta-regression analyses
4. Highlight existing data gaps and surveillance limitations to inform future research and support integration of GETV monitoring into global One Health surveillance programs.

By providing the first systematic and quantitative summary of GETV seroprevalence in animals, this study contributes essential baseline data for risk assessment, strengthens understanding of virus distribution and host range, and underscores the necessity for harmonized global surveillance and diagnostic standardization.

## MATERIALS AND METHODS

### Ethical approval

The systematic review and meta-analysis were performed in accordance with the Preferred Reporting Items for Systematic Reviews and Meta-Analyses (PRISMA) 2020 guidelines [12].

### Study period and location

The data were sourced, extracted, and analysed from April 2025 to June 2025 by researchers at the Universiti Sains Malaysia.

### Protocol registration and reporting framework

Preliminary searches were conducted using relevant keywords across multiple scientific databases to determine whether any meta-analyses on the global seroprevalence of GETV had previously been published. As no such studies were found, the review protocol was registered in the Open Science Framework (Registration ID: [doi.org/10.17605/OSF.IO/4YS2C]) to ensure methodological transparency and reproducibility.

### Search strategy

A comprehensive literature search was performed across major electronic databases, Scopus, PubMed, ScienceDirect, and Web of Science, to identify peer-reviewed studies reporting the seroprevalence of GETV in animals. No restrictions were applied regarding publication date or geographic region to maximize the retrieval of relevant literature.

The main search terms included combinations of “Getah virus,” “GETV,” and “Getah fever.” The exact search strings used for each database are provided in Supplementary File S1. The final database search covered all records available up to April 16, 2025. All identified citations were compiled in Mendeley, and duplicate entries were removed. The remaining unique records were screened using predefined eligibility criteria. In addition, reference lists of included articles were manually reviewed to identify further relevant studies.

### Eligibility criteria

The inclusion and exclusion of studies followed the Population–Exposure–Outcome (PEO) framework:

- Population: Animal populations tested for GETV antibodies
- Exposure: Infection or exposure to GETV
- Outcome: Reported seroprevalence of GETV infection.

Eligible studies were peer-reviewed articles published in English that reported quantitative seroprevalence data for GETV in specific animal populations. Studies were excluded if they:

1. Represented experimental or laboratory infection models,
2. Included humans or insect populations,
3. Used non-serological detection methods,
4. Reported outbreaks or epizootics without serological prevalence data,
5. Lacked accessible full texts or complete prevalence data, or
6. Were non-original research (reviews, case reports, conference abstracts, book chapters, opinion pieces, editorials, or letters).

### Screening and data extraction

Two independent reviewers screened all retrieved titles, abstracts, and full-text articles according to the eligibility criteria. Discrepancies between reviewers were resolved through consensus discussions.

For each eligible study, data were systematically extracted using a standardized Microsoft Excel template. Extracted variables included:

- First author and publication year,
- Study period and geographic location,
- Study population and animal species,
- Diagnostic method used for GETV detection,
- Number of seropositive samples, and
- Total number of animals tested.

### Statistical analysis

The pooled seroprevalence of GETV across all eligible studies was estimated using a DerSimonian–Laird random-effects model, with logit transformation applied to stabilize variance. The robustness of the pooled estimate was assessed through leave-one-out sensitivity analysis.

Publication bias was evaluated through funnel plot visualization and Egger’s regression test [13]. Between-study heterogeneity was quantified using Cochran’s Q test and I^2^ statistics, with I^2^ values of 25%, 50%, and 75% interpreted as low, moderate, and high heterogeneity, respectively [14, 15].

Subgroup analyses were conducted based on study location, host type, study period, and diagnostic method. To further explore sources of heterogeneity, univariate meta-regression was performed using the moment estimation method. All analyses were conducted using OpenMetaAnalyst (version 12; Brown University, USA) and Comprehensive Meta-Analysis (version 3; https://meta-analysis.com/) software, with statistical significance set at p < 0.05.

### Quality appraisal

The methodological quality of the included studies was independently assessed by both reviewers using the Joanna Briggs Institute (JBI) Critical Appraisal Checklist for prevalence studies [16] (Supplementary File S2). Each item was scored as “yes” (1) or “no” (0), generating a total possible score of 0–9. Studies scoring ≥7 were considered methodologically sound [17].

## RESULTS

### Literature search and study selection

**Figure 1:**
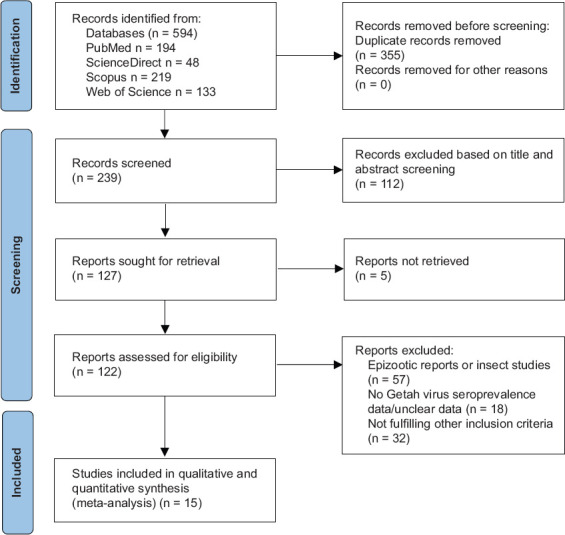
Database search and eligibility screening for Getah virus seroprevalence studies.

A comprehensive database search of Scopus, PubMed, ScienceDirect, and Web of Science initially yielded 594 records. After removing 355 duplicates, 239 unique records remained for screening (Figure 1). Titles and abstracts were reviewed to assess relevance to the study objectives and inclusion criteria, resulting in the exclusion of 112 records that did not meet eligibility requirements. Full-text evaluations were subsequently performed on the remaining studies, leading to the inclusion of 15 studies that satisfied all criteria for both the qualitative and quantitative analyses (Figure 1).

### Characteristics of included studies

Although the review aimed for a global perspective, all eligible studies were confined to Asian regions, indicating a substantial geographical knowledge gap. The included studies originated from five countries: China, Japan, Malaysia, South Korea, and Thailand, with the majority of data contributed by China and Japan.

Sample sizes varied considerably, ranging from 48 to 3,299 animals, encompassing both wild species (e.g., wild boars) and domestic animals, including pigs, horses, cattle, sheep, goats, ducks, and chickens. Among these, pigs and horses were the most frequently investigated species. The temporal coverage of the studies extended from 1962 to 2023, reflecting long-term but regionally restricted surveillance.

Serological detection methods included ELISA, HI test, and virus neutralization tests, highlighting methodological diversity across studies. Quality appraisal indicated that most studies were methodologically sound, achieving ≥7 points on the JBI quality checklist (Supplementary File S3). Key characteristics of the included studies are summarized in Table 1 [18–32].

**Table 1:** Overview of studies examined the seroprevalence of Getah virus.

Reference	Country	Sampling period	Method	Population	Sample size
Kuwata *et al*. [18]	Japan	2007-2016	ELISA	Wild boars	1048
Li *et al*. [19]	China	2015	PRNT	Domestic animals: chicken, duck, dairy cattle, pig, and beef cattle.	196
Liu *et al*. [20]	China	2022-2023	ELISA	Cattle	534
Liu *et al*. [21]	China	2018	MNT	Cattle	48
Matsumura *et al*. [22]	Japan	1973-1977	MNT	Horses	184
Park *et al*. [23]	South Korea	NR	MNT	Domestic pigs	670
Qiu *et al*. [24]	China	2021	ELISA	Horses	646
Rattanatumhi *et al*. [25]	Thailand	2017-2018	ELISA	Domestic pigs	1188
Shi *et al*. [26]	China	2017-2020	ELISA	Thoroughbred horses, local horses, goats, sheep, cattle, and pigs	3299
Simpson *et al*. [27]	Malaysia	1962-1964	Neutralization test	Pigs	272
Sugiura *et al.* [28]	Japan	1997	HI test	Racehorses	1644
Sugiyama *et al*. [29]	Japan	2000-2001	HI test	Wild boars	90
Sun *et al*. [30]	China	2018	ELISA	Pigs	133
Takeishi *et al*. [31]	Japan	2019-2020	PRNT	Noma horses	77
Zhong *et al*. [32]	China	2018-2020	VNT	Horses	182

ELISA = Enzyme-linked immunosorbent assay, HI = Hemagglutination inhibition, MNT = Microneutralization test, NR = Not reported, PRNT = Plaque reduction neutralization test, VNT = Virus neutralization test.

### Pooled seroprevalence of GETV in animals

**Figure 2:**
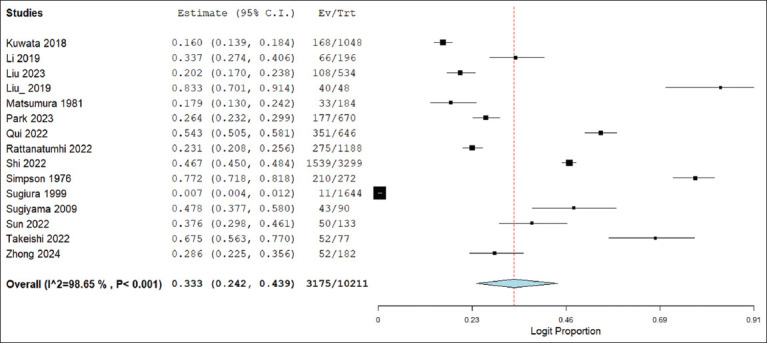
Forest plot of the pooled seroprevalence of the Getah virus in animals. The pooled GETV seroprevalence across studies was estimated using a DerSimonian–Laird random-effects model. C.I. = Confidence interval, I^2^ = Heterogeneity value.

Using a random-effects model, the pooled seroprevalence of GETV across all studies was estimated at 33.3% (95% confidence interval [CI]: 24.2–43.9), with significant heterogeneity (Q = 1033.881, I^2^ = 98.65%, p < 0.001). The pooled estimate and individual study weights are illustrated in the forest plot (Figure 2).

**Figure 3:**
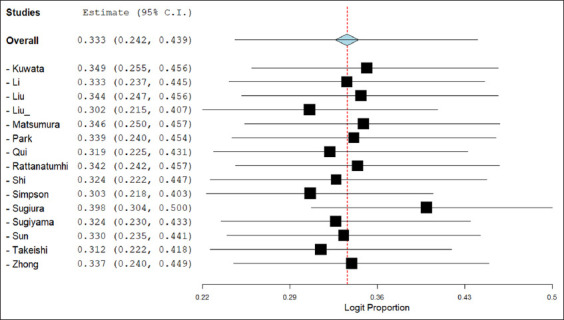
Leave-one-out sensitivity analysis of the pooled seroprevalence of the Getah virus in animals. The analysis was conducted by sequentially removing each study and recalculating the overall estimate using a DerSimonian–Laird random-effects model. C.I. = Confidence interval.

A leave-one-out sensitivity analysis was conducted to assess the robustness of the pooled estimate. Exclusion of individual studies yielded prevalence values ranging between 30.2% (when Liu *et al*. [21] was removed) and 39.8% (when Sugiura and Shimada [28] was excluded) (Figure 3), indicating the overall estimate was relatively stable and not disproportionately influenced by any single study.

**Figure 4:**
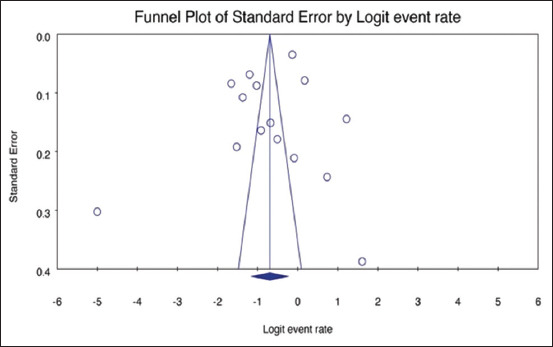
Funnel plot of the studies on the seroprevalence of Getah virus in animals (Egger’s p = 0.3968). The small circles in the plot indicate the studies included.

Potential publication bias was examined using funnel plot asymmetry and Egger’s regression test, which showed no statistically significant bias (p = 0.3968) (Figure 4).

### Subgroup analysis

Subgroup meta-analyses were performed to explore sources of heterogeneity and assess seroprevalence variations across geographic regions, study periods, host types, and diagnostic methods.

**Figure 5:**
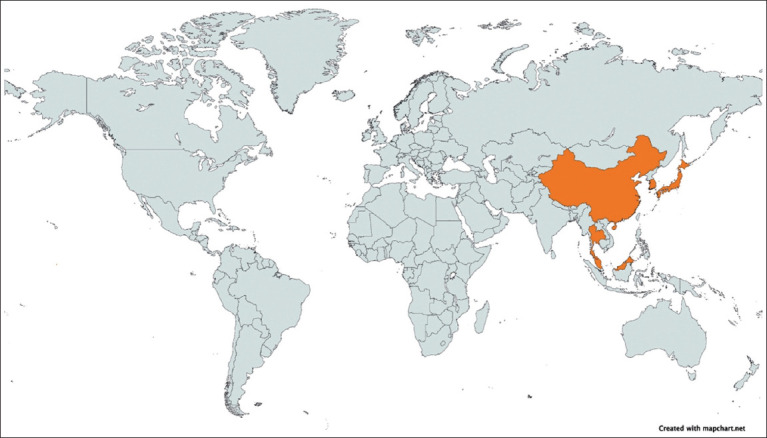
Geographic distribution of source countries for the seroprevalence data in this study, with contributing nations colored orange [Source: The map was generated using Mapchart (https://www.mapchart.net/world.html)].

- By country, all contributing studies were from Asia (Figure 5). Malaysia reported the highest seroprevalence at 77.2% (95% CI: 71.8–81.8) based on a single study, followed by China (41.8%, 95% CI: 31.5–52.7), while Japan showed the lowest pooled rate (18.3%, 95% CI: 5.7–45.4) (Table 2).
- By study period, seroprevalence was highest between 2000 and 2009 (47.8%, 95% CI: 37.7–58.0) and lowest before 1990 (14.7%, 95% CI: 0.7–80.7).
- By host category, domestic animals showed a slightly higher pooled prevalence (34.0%, 95% CI: 24.4–45.1) compared with wild animals (29.2%, 95% CI: 8.2–65.7). Among domestic species, pigs (43.1%, 95% CI: 27.4–60.4) and cattle (43.2%, 95% CI: 24.9–63.6) had the highest rates, followed by horses (28.5%, 95% CI: 12.6–52.6).
- By diagnostic method, studies using virus neutralization tests reported the highest seroprevalence (47.3%, 95% CI: 28.7–66.6), followed by ELISA (31.4%, 95% CI: 19.8–45.8), while those using the HI test yielded the lowest estimate (7.3%, 95% CI: 0.1–90.7).

**Table 2:** Seroprevalence of Getah virus in different subgroups of animals.

subgroups	Number of studies conducted	Prevalence (%)	95% CI	The heterogeneity test		

				Q	I^2^ (%)	p-value
Study location						
China	7	41.8	31.5–52.7	197.312	96.96	<0.001
Japan	5	18.3	5.7–45.4	265.824	98.50	<0.001
Malaysia	1	77.2	71.8–81.8	NA	NA	NA
South Korea	1	26.4	23.2–29.9	NA	NA	NA
Thailand	1	23.1	20.8–25.6	NA	NA	NA
Study period						
<1990	3	14.7	0.7–80.7	390.758	99.49	<0.001
2000–2009	1	47.8	37.7–58.0	NA	NA	NA
2010–2019	7	44.4	32.2–57.3	253.574	97.63	<0.001
>2019	2	35.5	10.8–71.5	133.954	99.25	<0.001
The animal category						
Domestic animals	13	34.0	24.4–45.1	830.812	98.56	<0.001
Wild animals	2	29.2	8.2–65.7	47.568	97.9	<0.001
Animal type						
Cattle	4	43.2	24.9–63.6	72.465	95.86	<0.001
Horses	6	28.5	12.6–52.6	561.135	99.11	<0.001
Pigs	6	43.1	27.4–60.4	303.001	98.35	<0.001
Wild boars	2	29.2	8.2–65.7	47.568	97.90	<0.001
Method of detection						
ELISA	6	31.4	19.8–45.8	539.925	99.07	<0.001
HI test	2	7.3	0.1–90.7	177.291	99.44	<0.001
Neutralization test	7	47.3	28.7–66.6	268.750	97.99	<0.001

CI = Confidence interval, ELISA = Enzyme-linked immunosorbent assay, NA = Not applicable, HI = Hemagglutination inhibition, Q = Cochran’s Q statistic.

All subgroups demonstrated high heterogeneity across studies (I^2^ > 95%) (Table 2; Supplementary File S4, Figures S1-S8).

### Meta-regression analysis

To further explore sources of heterogeneity, a univariate meta-regression was performed.

- Study location and detection method significantly contributed to between-study heterogeneity, whereas animal category did not. Location explained approximately 15% of the variance (Q = 12.08, df = 4, p = 0.017). Studies from Japan reported significantly lower seroprevalence compared with those from China (Coefficient = –1.169, 95% CI: –2.118 to –0.221, p = 0.015), while studies from Malaysia, although higher, did not reach statistical significance (p = 0.080). No significant differences were found between South Korea or Thailand, and China.
- Regarding diagnostic methods, studies employing the HI test reported significantly lower seroprevalence compared with those using the neutralization test (Coefficient = –2.363, 95% CI: –3.852 to –0.875, p = 0.002). Differences between ELISA and neutralization tests were not statistically significant (p = 0.198).

These findings indicate that both regional differences and diagnostic variation accounted for a measurable portion of the observed heterogeneity (Table 3).

**Table 3:** Univariate meta-regression analyses of heterogeneity-affected factors.

Variable	Number of studies conducted	Coefficient	95% CI	p-value	The test of the Model			R^2^ analog

					Q	d	p	
Study location	15				12.08	4	0.017	0.15
China (Reference)								
Japan		−1.169	−2.118–−0.221	0.015				
Malaysia		1.524	−0.184–3.231	0.080				
South Korea		−0.721	−2.413–0.972	0.404				
Thailand		−0.896	−2.585–0.793	0.298				
The animal category	15				0.12	1	0.729	0.04
Domestic animals (Reference)								
Wild animals		−0.229	−1.519–1.062	0.729				
Method of detection	15				9.78	2	0.008	0.00
Neutralization test (Reference)								
ELISA		−0.665	−1.677–0.347	0.198				
HI test		−2.363	−3.852–−0.875	0.002				

CI = Confidence interval, ELISA = Enzyme-linked immunosorbent assay, HI = Hemagglutination inhibition.

## DISCUSSION

### Global seroprevalence and epidemiological significance

The present meta-analysis estimated a pooled seroprevalence of 33.3% for GETV across both domestic and wild animal populations. This relatively high prevalence underscores the extensive circulation of GETV among diverse animal hosts and reinforces its potential importance to veterinary and possibly zoonotic health. The seroprevalence observed in this review exceeds that reported for other major arboviruses in animal hosts, for instance, chikungunya virus (17%) and Zika virus (6%) in non-human primates [10], and West Nile virus (8%) in equids across Europe [11].

Such a comparatively higher prevalence highlights GETV as a neglected but potentially emerging arbovirus, warranting enhanced global attention. Despite the lack of confirmed human infections, the widespread exposure in animals strengthens the rationale for integrating GETV surveillance within broader vector-borne disease monitoring programs under the One Health framework.

### Geographical patterns and regional disparities

Although this review imposed no regional restrictions, all 15 eligible studies were conducted in Asia, emphasizing the region’s central role in current knowledge of GETV ecology. The absence of reports from Africa, Europe, and the Americas indicates significant surveillance gaps.

Among the represented countries, Malaysia reported the highest seroprevalence (77.2%), although this estimate was based on a single study conducted prior to 1990. While the high value suggests intense viral exposure or sustained transmission in Malaysian animal populations [33, 34], reliance on a single study limits its generalizability and may not accurately reflect the current epidemiological situation, highlighting the need for updated investigations. China exhibited a substantial pooled seroprevalence of 41.8%, indicating widespread exposure to the virus and possibly necessitating more intensive surveillance efforts. Conversely, Japan recorded the lowest pooled seroprevalence (18.3%). These regional variations likely reflect ecological, environmental, and entomological factors, such as mosquito species diversity, climate variation, deforestation, and urbanization, that influence arbovirus transmission dynamics [35, 36].

### Temporal trends in seroprevalence

Temporal stratification revealed notable fluctuations in GETV seroprevalence across decades. The highest prevalence (47.8%) occurred between 2000 and 2009, although this was based on a single study, suggesting increased viral activity or enhanced diagnostic detection during that period. In contrast, studies conducted before 1990 reported the lowest prevalence (14.7%), possibly due to reduced virus circulation or limited awareness and testing capabilities.

These findings highlight the importance of longitudinal surveillance to capture evolving trends in virus activity and to assess the influence of environmental and anthropogenic factors such as climate change and land-use modification on vector ecology [37–39]. Periodic re-evaluation of global data is essential to maintain accurate risk assessments and guide responsive control strategies.

### Host-specific patterns and transmission dynamics

The analysis demonstrated comparable exposure levels between domestic (34.0%) and wild animals (29.2%), suggesting widespread virus circulation across ecological boundaries. Domestic species, due to their proximity to humans and increased vector contact, likely play a more prominent role in viral maintenance and transmission [40, 41].

Among domestic animals, pigs (43.1%) and cattle (43.2%) exhibited the highest seroprevalence rates, implying heightened susceptibility or frequent exposure in endemic regions [4]. Pigs, in particular, are recognized as amplifying hosts for multiple arboviruses, enhancing transmission potential to other animals and possibly to humans [42, 43]. Similarly, cattle may act as sentinel species, reflecting virus activity in the environment due to their outdoor rearing and continuous exposure to mosquito vectors.

In contrast, horses displayed a lower seroprevalence (28.5%), suggesting reduced exposure or different susceptibility mechanisms. However, the occurrence of clinically significant GETV outbreaks in equine populations, manifesting as fever, rash, and hind limb edema, makes horses an epidemiologically important species that warrants continued surveillance and preventive measures [44].

### Influence of diagnostic methods

Substantial variation in reported seroprevalence was observed across diagnostic techniques. Studies using virus-neutralization tests reported the highest pooled estimate (47.3%), consistent with the test’s superior specificity and functional relevance as the gold standard for detecting neutralizing antibodies [45, 46].

The ELISA-based studies yielded moderate seroprevalence (31.4%), reflecting its sensitivity and scalability for population-level screening [47, 48]. However, ELISA can detect cross-reactive antibodies from related alphaviruses, potentially causing overestimation of prevalence [49, 50]. Conversely, the HI test produced the lowest seroprevalence (7.3%), which may be due to its comparatively lower sensitivity and susceptibility to antigenic mismatch [51].

These discrepancies highlight the need for standardized diagnostic protocols to improve data comparability across studies. Future serosurveillance should prioritize neutralization-based assays for confirmatory testing while using ELISA for large-scale screening. Consistent assay use and harmonized cutoff criteria would significantly reduce heterogeneity and strengthen epidemiological interpretations.

### Strengths and limitations

This study represents the first systematic review and meta-analysis to estimate the global seroprevalence of GETV in animal populations. The strengths include an unrestricted search strategy, methodological rigor, and comprehensive inclusion of data from multiple host species and detection methods.

However, several limitations should be acknowledged. All eligible studies originated exclusively from Asia, which limits the generalizability of the findings to other continents. The high heterogeneity (I^2^ = 98.65%) among studies, along with the reliance on some older or single-country datasets, constrain interpretive precision. In addition, some included studies employed the HI test, which may underestimate seroprevalence due to antigenic mismatches with circulating strains. Despite these limitations, the present review provides critical baseline evidence for understanding GETV epidemiology and underscores the importance of expanding surveillance to underrepresented regions and host species.

## CONCLUSION

This systematic review and meta-analysis estimated the global pooled seroprevalence of GETV in animals at 33.3% (95% CI: 24.2–43.9), confirming widespread exposure among diverse hosts, particularly domestic livestock such as pigs and cattle. The consistently high rates in these species indicate their potential role as sentinel or amplifying hosts, contributing to virus maintenance in endemic regions. Geographically, all available data were confined to Asia, with the highest seroprevalence recorded in Malaysia (77.2%), followed by China (41.8%) and Japan (18.3%), highlighting both the regional concentration of evidence and the absence of surveillance data from other parts of the world. Meta-regression analysis further revealed that study location and diagnostic methodology significantly contributed to the observed heterogeneity, emphasizing the need for standardized testing protocols to improve global comparability of results.

From a One Health perspective, the extensive circulation of GETV among animal populations raises concern for potential zoonotic spillover, as serological evidence suggests occasional human exposure. Strengthening integrated surveillance systems that simultaneously monitor vectors, animals, and humans is therefore critical to detect and mitigate cross-species transmission risks.

Looking ahead, future research should expand surveillance efforts beyond Asia, adopt standardized high-specificity assays such as virus-neutralization tests, and conduct longitudinal and vector-based studies to elucidate transmission dynamics. Integrating veterinary, entomological, and human health monitoring within a unified One Health framework will be essential to enhance preparedness and response to this emerging arboviral threat.

In conclusion, this study provides the first comprehensive global evidence of GETV exposure in animal populations, highlighting its endemic presence in Asia and potential for wider emergence. These findings underscore the urgent need for enhanced surveillance, diagnostic harmonization, and cross-sectoral collaboration to strengthen early detection and containment of GETV within animal and public health systems.

## DATA AVAILABILITY

The supplementary data can be available from the corresponding author upon reasonable request.

## AUTHORS’ CONTRIBUTIONS

AAI and RHS: Conceptualized and designed the study, data collection, extraction, and processing, prepared and interpreted the results, and drafted and edited the manuscript. AAI: Data analysis. RHS: Supervised the study. Both authors have reviewed and approved the final version of the manuscript.
